# Trace Element Interactions, Inflammatory Signaling, and Male Sex Implicated in Reduced Growth Following Excess Oral Iron Supplementation in Pre-Weanling Rats

**DOI:** 10.3390/nu14193913

**Published:** 2022-09-21

**Authors:** Shasta A. McMillen, Eric B. Nonnecke, Bo Lönnerdal

**Affiliations:** Department of Nutrition, University of California, Davis, CA 95616, USA

**Keywords:** iron supplementation, iron toxicity, infants, rat model, trace mineral interactions, inflammation

## Abstract

Iron supplements are frequently provided to infants in high-income countries despite low incidence of iron deficiency. There is growing concern regarding adverse health and development outcomes of excess iron provision in early life. Excess iron may directly damage developing organs through the formation of reactive oxygen species, alter systemic inflammatory signaling, and/or dysregulate trace mineral metabolism. To better characterize the in vivo effects of excess iron on development, we utilized a pre-weanling rat pup model. Lewis rat litters were culled to eight pups (four males and four females) and randomly assigned to daily supplementation groups receiving either vehicle control (CON; 10% *w/v* sucrose solution) or ferrous sulfate (FS) iron at one of the following doses: 10, 30, or 90 mg iron/kg body weight—FS-10, FS-30, and FS-90, respectively—from postnatal day (PD) 2 through 9. FS-90 litters, but not FS-30 or FS-10, failed to thrive compared to CON litters and had smaller brains on PD 10. Among the groups, FS-90 liver iron levels were highest, as were white blood cell counts. Compared to CON, circulating MCP-1 and liver zinc were increased in FS-90 pups, whereas liver copper was decreased. Growth defects due to excess FS provision in pre-weanling rats may be related to liver injury, inflammation, and altered trace mineral metabolism.

## 1. Introduction

Iron deficiency (ID) is a common micronutrient deficiency that underlies approximately half of all anemia cases in infants, children, and adults worldwide [1,2,3]. Infants are particularly at risk for ID, which, in addition to causing anemia, can disrupt cognitive development and increase risk for infection [3,4,5]. The American Academy of Pediatrics recommends supplemental iron for all infants to prevent ID [6]. Supplemental iron can also be harmful if provided beyond physiological requirements [7,8,9,10,11]. A growing number of studies report adverse effects of iron provision in iron-replete infants [12,13,14,15,16], but the mechanisms behind iron toxicity in early life are not well understood. Recently, the 2020 Dietary Guidelines Advisory Committee proposed that improving the efficacy of supplemental iron provision in infants necessitates that future research investigate the mechanisms underlying adverse health and development effects of iron [7,8,17,18,19].

The effects of excess iron on growth and neurodevelopment during early life remain unclear. Deleterious effects of iron provision on anthropomorphic measures of growth in iron-replete infants have been observed; however, associated defects in nervous system development, including cognitive delay, have been observed independently of delayed growth [9,12,13,20,21,22,23]. Such inconsistences are likely underscored by variation in the form and/or dose of iron provided in the study and interactions with baseline iron status or baseline nutritional status. Iron potentiates the formation of reactive oxygen species that disrupt cellular events required for normal organ development. Furthermore, supplemental iron can dysregulate cellular zinc and copper pools, exacerbating the deleterious outcomes associated with iron alone [13,24,25,26]. 

Ferrous sulfate (FS) is an inexpensive form of iron present in both iron drops and infant formulas [27]. Excess FS can have adverse effects on growth and cognitive development [12,14,28,29,30], but the relationship between FS dose and infant development is not well-established [31]. Using an iron supplementation model in pre-weanling rats, we investigated health and development outcomes of daily FS supplementation at three concentrations: 10, 30, and 90 mg iron/kg body weight (BW). FS supplementation at 90 mg iron/kg BW reduced growth, increased inflammation, and reduced brain size. Our results indicate that excess FS disrupts postnatal growth and neurodevelopment, and these outcomes may be related to liver iron loading, systemic inflammation, and trace element interactions. 

## 2. Materials and Methods

### 2.1. Animal Experiments

Animal experiments were approved by the University of California Davis Institutional Animal Care and Use Committee. Adult male and female Lewis rats (Charles River Laboratories, Wilmington, MA, USA) were housed under standard conditions for the duration of the study: in clear polycarbonate hanging cages in a room with constant temperature (22 °C), humidity (63%), and 12 h light/dark cycles. Animals were provided ad libitum access to 18% protein rodent chow (200 mg Fe/kg diet; 2018, Teklad Diets, Madison, WI, USA). Nulliparous females—8–10 weeks old—were used for generating experimental litters. On postnatal day (PD) 2, litters were culled to sex-matched litters of 8 pups to standardize milk-availability, cross-fostering from litters born within 24 h period, as necessary. Litters (n = 12) were randomly assigned supplementation groups—the number of pups from each treatment group as well as the number of litters represented are indicated in the Methods and Results. Pups from multiple litters receiving the same treatment were assessed to account for litter effect. Groups were as follows: 10, 30, and 90 mg iron/kg body weight ferrous sulfate heptahydrate (Cat#215422-250G, Sigma-Aldrich, St. Louis, MO, USA), or vehicle control (CON; 10% *w/v* sucrose). The lowest dose (10 mg iron/kg) represented of daily iron intake by formula-fed infants [32]. The 30 and 90 mg doses represented excess iron provision; and were below toxicity levels reported in adult rats [33]. Oral iron was administered once daily PD 2–9, in the afternoon by hand-pipetting. Body weight (BW) was recorded on PD 2, 4, 6, 8, and 10 (study end; day of necropsy). Supplement volume was calculated according to body weight and ranged from 8–60 µL.

### 2.2. Necropsy and Hematology

On PD 10, litters were separated from dams and fasted for 4–6 h. Pups were weighed and euthanized by decapitation under deep anesthesia (100 mg ketamine × 10 mg xylazine/kg BW). Whole blood was collected into EDTA-treated tubes (Greiner Bio-One, Monroe, NC, USA) and held at 4 °C until processed. Whole brains were removed, weighed, and promptly dissected into four regions: prefrontal cortex (PFC), striatum (ST), hippocampus (HP), and cerebellum (CE). All brains were dissected by the same researcher for consistency.

Complete blood counts (CBC; n = 3–4/group, 2 litters/group) were performed by the UC Davis School of Veterinary Medicine Clinical Pathology Laboratory within 24 h of blood collection. 

### 2.3. Non-Heme Iron

Non-heme iron concentrations (n = 14–18/group, 3 litters/group for each tissue) of liver, kidney, spleen, and the four brain regions were determined by bathophenanthroline method [34]. 

### 2.4. Histopathology

Freshly isolated livers (n = 6/group, 3 litters/group) were immersion-fixed in 4% (*w*/*v*) paraformaldehyde (PFA) for 24 h at 4 °C, then washed in three changes of 1 × PBS and stored in 70% ethanol at 4 °C. Fixed livers were embedded in paraffin using standard protocols. The UC Davis School of Veterinary Medicine Anatomic Pathology Laboratory completed sectioning and staining. Liver sections were stained with Perls’ Prussian Blue with nuclear fast red counterstain for iron detection, as well as Masson’s Trichrome for evaluation of liver injury and inflammation. 

Masson’s Trichrome-stained liver slides were scored for injury and inflammation in a blinded fashion using a non-alcoholic fatty liver scoring system for rodents [35]. A veterinary pathologist at the UC Davis School of Veterinary Medicine Comparative Pathology Laboratory assessed and scored steatosis (micro- or macro-vesicular), inflammation, fibrosis, and necrosis. Scores for steatosis, fibrosis, and necrosis were assigned as follows: 0, parameter absent; 1, <10% affected area; 2, 10–25% affected area; 3, 26–50% affected area; 4, >50% affected area. Distribution of steatosis, fibrosis, and necrosis were classified as random, centrilobular, midzonal, periportal, or diffuse (all zones equally affected). Inflammation was scored according to number of inflammatory foci/field as follows: 0 = parameter absent, 1 = minimal, scattered, rare (<1 per 20 × field), 2 = mild (<2 per 20 × field), 3 = moderate (2–4 per 20 × field), and 4 = severe (>4 per 20 × field). Presence or absence of degeneration, hypertrophy, and oval cell/biliary hyperplasia was recorded. 

### 2.5. Liver Gene Expression

At necropsy, tissues were collected into RNA*later* ^®^ (Thermo Fisher Scientific™, Waltham, MA, USA), incubated for 24 h at 4 °C, and then stored at −20 °C until RNA extraction. The TRIzol method (Invitrogen™, Carlsbad, CA, USA) was used to extract RNA from liver tissue, as outlined by the manufacturer’s protocol. Reverse transcription of total RNA to cDNA was performed using the High-Capacity cDNA Archive Kit (Cat#4374966, Applied Biosystems™, Foster City, CA, USA), and stored in EB buffer at 4 °C. Real-time PCR reactions were performed using iTaq Universal SYBR Green Supermix (Cat#1725121, Bio-Rad, Hercules, CA, USA), and Bio-Rad CFX96 Real-Time machine. Primers for qRT-PCR were: *Actb* forward, 3′-GAAATCGTGCGTGACATTAAAGAG-5′; *Actb* reverse, 3′-GCGGCAGTGGCCATCTC-5′ [36]; *Hamp* forward 3′-GCTGCCTGTCTCCTGCTTCT-5′; *Hamp* reverse 3′-CTGCAGAGCCGTAGTCTGTCTCGTC-5′ [37]. Data analysis of the qRT-PCR results was performed using the delta/delta CT (2^−∆∆Ct^) method, with *Actb* serving as the housekeeping gene (n = 13–18/group, 3 litters/group). 

For RNA-seq analysis, RNA (*n* = 3 males/group; 1 male from each litter) was purified with the RNA Clean & Concentrator™ kit with DNase (Zymo Research Corporation, Irvine, CA, USA) and submitted to the DNA Technologies and Expression Analysis Core Laboratory at the UC Davis Genome Center. RNA quality was assessed by LabChip^®^ GX prior to library construction. The library was sequenced on an Illumina HiSeq 4000 platform for 3’-Tag-Seq gene expression profiling. Read count matrices were constructed using salmon [38] and a reference *Rattus norvegicus* transcriptome (Ensembl genome assembly mRatBN7.2, release 106) [39].

### 2.6. Serum Chemokine/Cytokine Array

Fresh whole blood was incubated in sterile tubes at room temperature for 30 min, and then centrifuged at 300 rcf for 15 min to isolate serum. Sera were diluted 1:1 in 1 × PBS and shipped on dry ice to Eve Technologies Corporation (Calgary, AB, Canada). Array services were used (Cat#RD27) for quantifying 27 total chemokines and cytokines (n = 10/group, 3 litters/group): CCL11, EGF, CX3CL1, IFNγ,IL-1α, IL-1β, IL-2, IL-4, IL-5, IL-6, IL-10, IL-12(p70), IL-13, IL-17A, IL-18, IP-10, CXCL1, TNFα, G-CSF, GM-CSF, CCL2, Leptin, CXCL5, CCL3, CXCL2, CCL5, and VEGF.

### 2.7. Zinc and Copper

Zinc and copper concentrations (n = 9–18/group, 3 litters/group for each tissue) were determined in liver, kidney, spleen, and brain regions—PFC, ST, HP, and CE—by atomic absorption spectrometry using methods described previously [32]. 

### 2.8. Statistical Analysis

Statistical analysis and plotting were performed with GraphPad Prism (v9.3.1). The study was designed to consider the variation between litters, as well as within litters, while testing for differences among groups. When testing for differences in weight gain among groups, pup body weights were averaged by litter and litters were treated as biological replicates. A two-way repeated measure ANOVA was applied to test for effects of time and supplementation group on litter weight gain and post hoc Tukey’s test detected differences between groups. Samples for all other outcomes besides weight gain were taken randomly from multiple litters in the same group to capture litter variation. The sample size of pups and number of litters represented in each of the outcomes are listed in the corresponding methods above. All datasets were checked for normality using the Shapiro–Wilks test. Kruskal–Wallis and Dunn’s tests were used for finding overall group effects and differences between groups, respectively, in non-parametric data. *p* < 0.05 determined significance. 

For analysis of RNA-seq data, read count matrices that had been constructed in salmon [38] were imported into R [40] with Tximeta [41] for differential gene expression analysis of liver tissue according to iron dose group using DESeq2 [42]. Comparisons of expression between groups were adjusted for false discovery rate (FDR) and *p*-values < 0.05 were considered significant. 

## 3. Results

### 3.1. Excess FS Disrupts Growth

**Figure 1:**
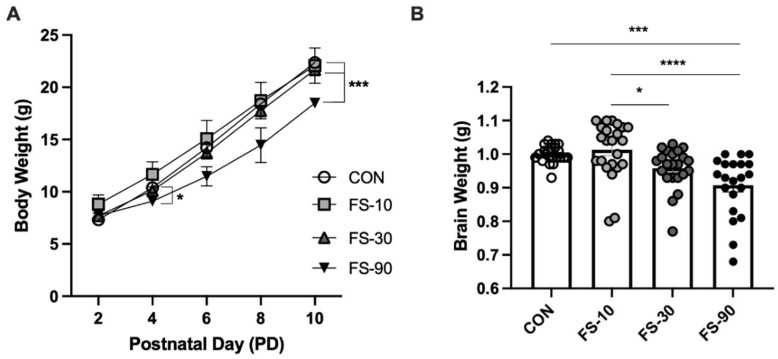
Growth and development are disrupted by excess ferrous sulfate (FS) iron supplementation in pre-weanling rats. (**A**) Postnatal growth was impaired in FS-90 supplemented animals; postnatal day (PD) 2–10. Body weight of pups (n = 21–24/group, 3 litters/group) was measured every two days (means ± SEM). (**B**) Brain weight at PD 10 was reduced the in FS-90 group vs. CON. Biological replicates (n = 21–24/group, 3 litters/group) are shown as individual data points with mean ± SEM. Vehicle control (CON); ferrous sulfate (FS)-10 (10 mg iron/kg BW); FS-30 (30 mg iron/kg BW); FS-90 (90 mg iron/kg BW). *p*-value summary: *, *p* < 0.05; ***, *p* < 0.001; ****, *p* < 0.0001.

Average pup BW increased over time in all groups but was negatively impacted by FS-90 supplementation (Figure 1A). The BW of pups in FS-10 and FS-30 groups mirrored CON. However, in the FS-90 group BW was approximately 10% lower than CON by PD 4 (*p* < 0.05), and 17% lower by PD 10 (*p* < 0.001). Additionally, FS-90 brains weighed 10% less than CON and FS-10 brains at PD 10 (Figure 1B, *p* < 0.001). 

When stratified by sex, BW at PD 10 was lower only in FS-90 males compared to FS-10 and FS-30 males, an effect that was sex-specific (Figure S1A,B). Similarly, brain weight of FS-90 supplemented males but not females was lower than CON and FS-10 males (Figure S1C,D). 

### 3.2. Liver Iron Loading from FS Dosing

**Figure 2:**
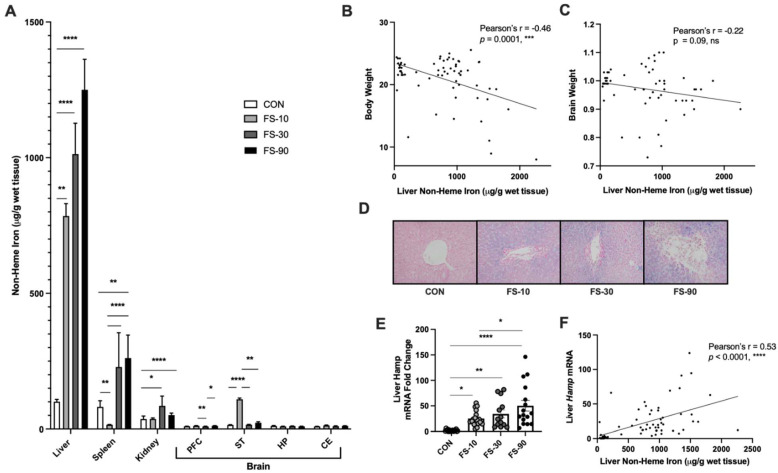
Liver iron loading and hepcidin expression increased with iron dose. (**A**) Tissue non-heme iron concentration on PD 10 following 8 days of FS supplementation; PD 2–9. Data for each tissue (n = 14–18/group, 3 litters/group) are represented as mean ± SEM. (**B**) Correlation analysis of liver non-heme iron concentration vs. PD 10 body weight and (**C**) brain weight. (**D**) Representative images liver sections stained with Perls’ Prussian blue (n = 6 per group). (**E**) Fold-change in liver *Hamp* mRNA expression, assessed by RT-qPCR. Biological replicates (n = 13–18/group, 3 litters/group) are shown as individual data points with mean ± SEM. (**F**) Correlation analysis of liver non-heme iron concentration vs. liver *Hamp* expression. *p*-value summary: *, *p* < 0.05; **, *p* < 0.01; ***, *p* < 0.001; ****, *p* < 0.0001; ns = non-significant.

To identify tissues impacted by iron loading following excess iron supplementation in pre-weanling rats, non-heme iron levels were quantified in liver, kidney, spleen, and four brain regions, including the prefrontal cortex (PFC), striatum (ST), hippocampus (HP), and cerebellum (CE). Liver iron concentration increased in response to FS (*p* < 0.0001; Figure 2A). Relative to CON, liver iron increased approximately 800% in the FS-10 group, 900% in the FS-30 group, and 1100% in the FS-90 group; however, there was no significant difference in liver iron concentration among FS supplemented groups. In spleen, iron levels were reduced approximately 80% in the FS-10 group (*p* < 0.01) but increased 220% in the FS-90 group (*p* < 0.01). Iron levels in kidney and all four brain regions were unmodified by supplemental iron at any dose. Iron deposition was undetectable at 40x objective in CON liver sections stained for Perls’ Prussian blue for iron (Figure 2D) but was apparent even at low magnification (10x objective) in FS-group livers. Hepatic iron concentration was inversely correlated to BW (*p* = 0.0001; Figure 2B) but independent of brain weight (*p* = 0.09; Figure 2C). These results suggest elevated liver iron stores are inversely associated with growth, including weight gain in pre-weanling rats. 

Liver hepcidin (*Hamp*) mRNA expression was induced in all FS groups, with the highest relative expression observed in FS-90 liver (~50-fold, *p* < 0.0001). Hepatic non-heme iron was significantly correlated to hepcidin expression (Figure 2E,F). Thus, hepcidin is inducible in the liver of pre-weanling rats and is reflective of iron loading, as observed in adult animals [43]. 

When data was stratified by sex, the spleen, kidney, and PFC non-heme iron concentrations were significantly increased by iron loading in males only (Figure S2A). In contrast, liver non-heme iron concentration was increased by FS in both male and female animals (Figure S2B). Iron concentration in CON tissues was similar between males and females apart from kidney tissue, where mean values were greater in females than male CON. Consistent between males and females, iron loading was most evident in the FS-90 livers, where it reached over 1300 μg iron/g tissue in FS-90 groups (Figure S2). There was no observed sex effect of liver *Hamp* expression.

### 3.3. Hematological Effects of Excess FS

Hemoglobin, hematocrit, red blood cell (RBC) count, mean corpuscular volume (MCH), and mean cell hemoglobin concentration (MCHC) were unaffected by iron dose (CBC results shown in Table 1). However, MCH was altered by iron dose (*p* < 0.0001) and was elevated with FS-30 compared to FS-10. Although there was an overall effect of dose on RBC distribution width (RDW) (*p* = 0.008), the difference between the FS-30 and FS-90 groups and CON was not significant (0.08 > *p* > 0.05). 

Corrected WBC count was elevated in FS-90 compared to CON group (*p* < 0.05). FS supplementation dose influenced neutrophil count; however no clear effect was observed between CON and the highest (FS-90) iron dose (0.08 > *p* > 0.05). No effect of FS was observed on monocyte or lymphocyte counts. CBC analysis was not sufficiently powered to determine effects of sex.

### 3.4. Liver Histopathology 

**Figure 3:**
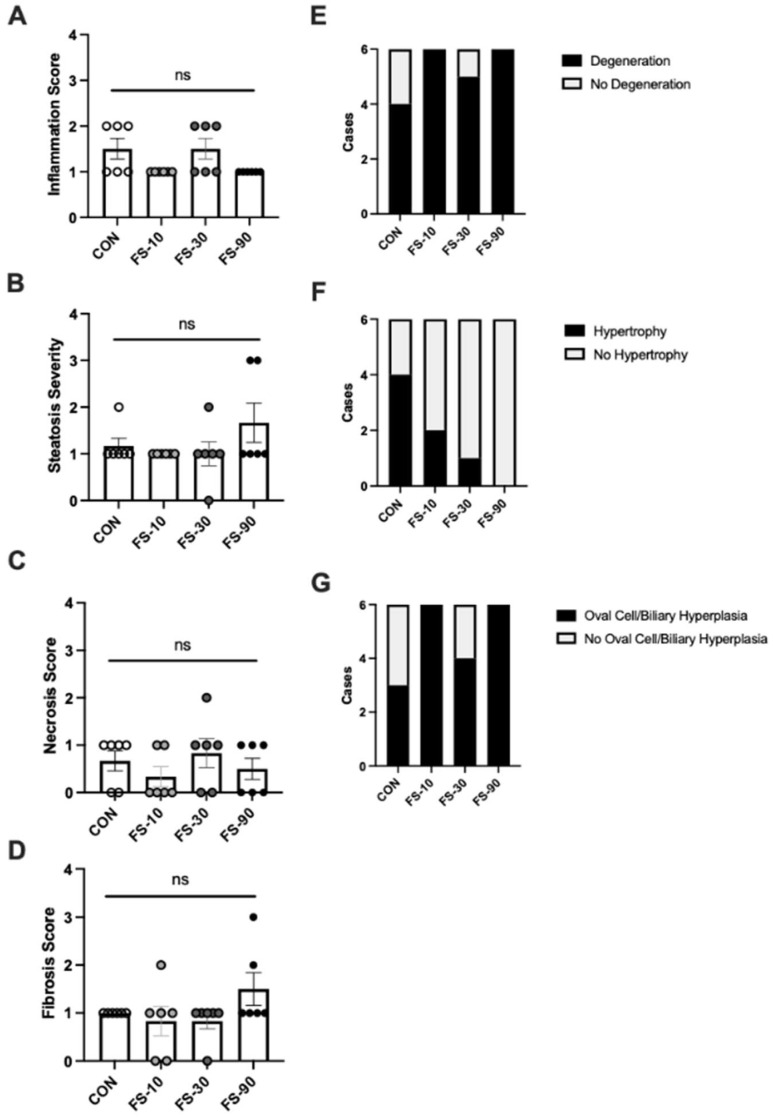
Hepatic inflammation and injury scores from histopathology following excess iron dosing. (**A**–**D**) Liver inflammation, steatosis, necrosis, and fibrosis severity scores. Biological replicates (n = 6/group) are shown as individual data points with mean ± SEM. (**E**–**G**) Histological assessment of degeneration, hypertrophy, and oval cell/biliary hyperplasia. Data represent number of cases per group. ns = non-significant.

Liver injury and inflammation (n = 6/group) was assessed by a pathologist in a blinded fashion (Figure 3). Inflammation was either mild or minimal, and there was no significant effect of FS dose on inflammation score. Randomly distributed microvesicular steatosis, periportal fibrosis, and necrosis scores were similar between groups. Degeneration and oval cell/biliary hyperplasia were present in all groups, and hypertrophy was detected in all groups except the FS-90 group; however, no significant differences were observed. There was no sex effect observed among groups in liver pathology outcomes. 

### 3.5. Chemokine and Cytokine Response

Serum fractalkine (CX3CL1), interferon gamma (IFNγ), GM-CSF (Granulocyte-macrophage stimulating factor), C-C motif chemokine 2 (CCL2), leptin, C-X-C motif chemokine 5 (CXCL5), CXCL2 (C-X-C motif chemokine 2), and vascular endothelial growth factor (VEGF) were modified by FS dose (Table 2). 

Fractalkine (CX3CL1), a chemokine expressed mainly by the central nervous system that plays an important role in early brain development [44], was reduced in FS-30 and FS-90 sera compared to CON (*p* = 0.0018). GM-CSF (Granulocyte-macrophage colony stimulating factor) and C-C motif chemokine 2 (CCL2), a pro-inflammatory chemokine, were elevated in FS-90 compared to FS-10 only (*p* < 0.05); differences observed between FS-90 and CON or FS-30 group means were not significant. Leptin an appetite-suppressing hormone that is upregulated by inflammation [45]. Leptin levels were elevated in the FS-30 group compared to FS-10 (*p* < 0.05). CXCL5, a neutrophil chemoattractant [46] and was reduced in FS-90 sera compared to CON (*p* < 0.05). Levels of CXCL2 (C-X-C motif chemokine 2) and vascular endothelial growth factor (VEGF) were lower in FS-30 sera compared to CON (*p* < 0.05), but there were no differences among the other dose groups. Chemokine and cytokine quantification was not sufficiently powered to test for sex effects. 

### 3.6. Alterations to Liver Zinc and Copper rr

**Figure 4:**
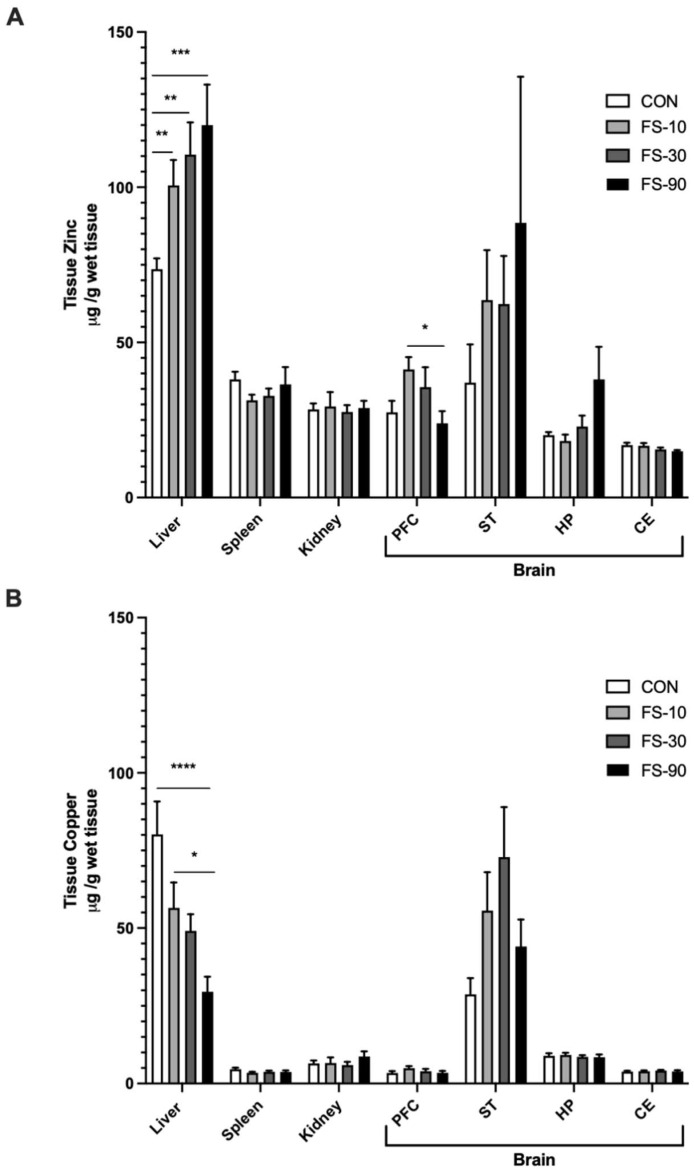
Hepatic zinc and copper levels are altered by excess iron. Tissue zinc (**A**) and copper (**B**) were determined using atomic absorption spectroscopy. Data (n = 9–18/group, 3 litters/group) are represented as mean ± SEM. *p*-value summary: *, *p* < 0.05; **, *p* < 0.01; ***, *p* < 0.001; ****, *p* < 0.0001.

Zinc and copper concentrations were analyzed in liver, spleen, kidney, PFC, ST, HP, and CE; however, a treatment effect was only observed in liver tissue (Figure 4). Liver zinc increased with all iron doses (*p* < 0.01), whereas liver copper was reduced in only FS-90 animals (*p* < 0.0001). 

When tissue zinc and copper content was stratified by sex, zinc concentration was similar between sexes across CON tissues (Figure S3A,B). In contrast, mean liver copper concentration was approximately 30% higher in female animals compared to males. Liver copper concentration in the FS-90 group was reduced to ~30 µg iron/g tissue in both male and female animals. 

### 3.7. Alterations to Liver Gene Expression

**Figure 5:**
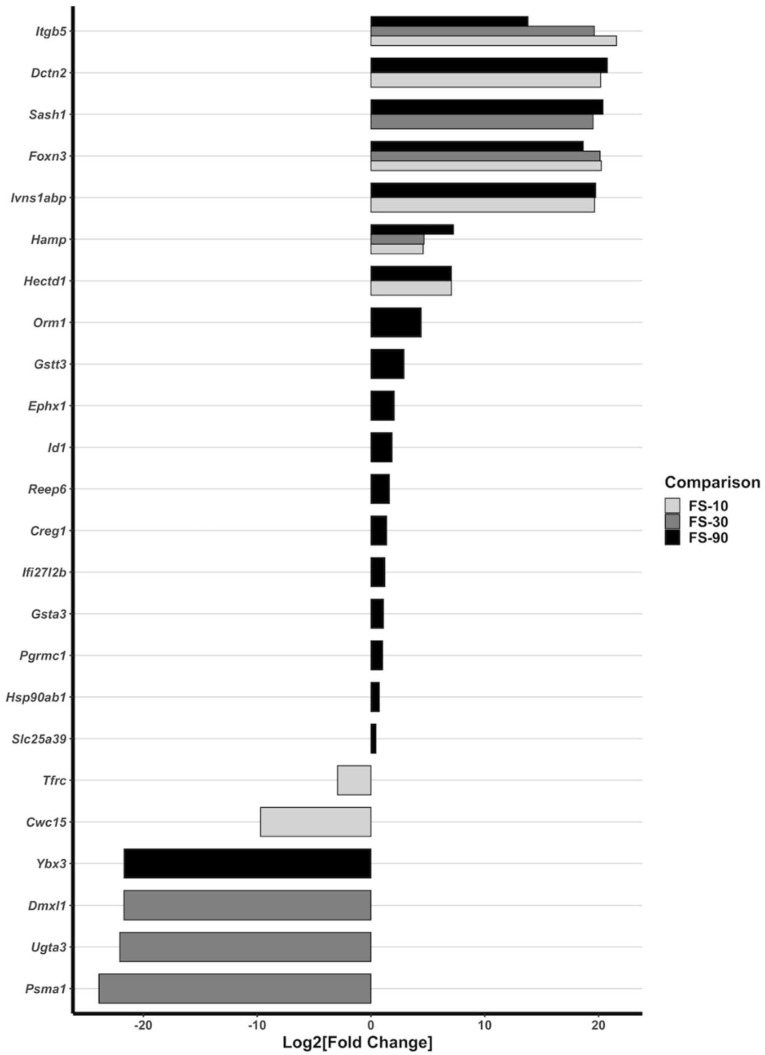
Hepatic mRNA expression is differentially modulated by FS supplementation. All genes determined to significantly different between CON and FS-10, FS-30, or FS-90 are displayed. Data is arranged on the *y*-axis in ascending order of expression magnitude relative to CON. Log2[Fold Change] values (*x*-axis) were determined with DESeq2, and FDR-adjusted *p*-values < 0.05 were considered significant.

Genes significantly altered by FS in liver are depicted in Figure 5, ordered in ascending magnitude. Detailed results are in Table S1. In total, 24 genes were up- or down-regulated in FS livers compared to CON. Consistent with real-time qPCR findings, *Hamp* mRNA was strongly up-regulated in all FS livers (*p* < 0.0001): ~20-fold in the FS-10 and FS-30 group, and ~150-fold in the FS-90 group. Of all up-regulated genes, *Itgb5* and *Foxn3* exhibited the largest degree of change due to FS. ln FS-10 and FS-30 livers, *Itgb5* expression was 10^6^-fold greater than in CON; by comparison, FS-90 liver *Itgb5* expression was 10^3^-fold greater than in CON. Similarly, FS-10 and FS-30 liver expression of *Foxn3* were 10^6^-fold greater than CON, while the change in FS-90 expression was 10^5^-fold greater than in CON. 

Other gene expression changes also depended on the dose of iron received. *Sash1* encodes SAM and SH3 domain-containing 1 protein and up-regulates the transcription of NFκB-dependent pro-inflammatory cytokines following TLR4 activation [47]; *Sash1* was the only gene up-regulated in both FS-30 and FS-90 but not FS-10 livers. *Sash1* may be involved in tumor suppression; induction of *Sash1* appears to be involved in NFκB-dependent apoptotic signaling [48]. In other cases, there was an unclear relationship between FS dose and modulation of gene expression. *Dctn2* (dynactin subunit 2), *Ivns1abp* (influenza virus NS1A-binding protein), and *Hectd1* (HECT domain E3 ubiquitin protein ligase 1) were strongly up-regulated in FS-10 and FS-90 livers but were not significantly changed in FS-30 livers. *Tfrc* (transferrin receptor) and *Cwc15* (CWC15 spliceosome-associated protein) were down-regulated in FS-10 livers, but not in FS-30 or FS-90 livers. Additional genes were up-regulated in FS-90 livers but not the lower dose groups, including *Orm1, Gstt3, Ephx1, Id1, Reep6, Creg1, Ifi27126, Gsta3, Pgrmc1, Hsp90ab1,* and *SIc25a39. Ybx3* (Y-box binding protein 3) was strongly down-regulated (10^−6^-fold CON expression) in FS-90 livers alone. *Dmxl1*, *Ugta3*, and *Psma1* were strongly down-regulated (10^−6^-fold CON expression) in FS-30 livers alone.

## 4. Discussion

Iron is essential for growth and metabolism but in excess can be toxic. Although beneficial to many infants, supplemental iron provided to those who are iron-replete can deleteriously affect growth and cognitive development [7,9]. Excess iron may interfere with growth through its accumulation in developing tissue and associated toxicity, including formation of reactive oxygen species. Furthermore, iron accumulation can disrupt the metabolism and transport of other trace minerals that are essential for normal growth. To investigate mechanisms of growth disruption, pre-weanling Lewis rats were supplemented from postnatal day (PD) 2 to 10 with excess FS iron doses (10, 30, or 90 mg iron/kg BW) or vehicle control (CON), and growth, tissue iron loading, systemic inflammation, and trace mineral status outcomes were assessed. Compared to CON, FS-90 litters exhibited impaired growth, an effect that was not observed in FS-10 or FS-30 litters. Liver iron loading was observed in all FS groups, where the highest levels were occurred in the FS-90 group. Delayed weight gain and lower brain weights observed in the FS-90 group may have been related to inflammatory signaling and altered trace mineral metabolism. Finally, the effect on brain weight observed in the FS-90 group did not correlated to liver or brain iron levels—suggesting that adverse long-term cognitive-behavioral effects of excess iron may not be related to early brain iron accumulation, as previously suspected.

Studies from our group and others show that intestinal iron absorption is elevated in pre-weanling animals [29,37,49,50], likely increasing susceptibility to iron toxicity. The LD50 for ferrous sulfate in adult rats is estimated to be 780–1100 mg iron/kg BW [51,52], where reduced body weight was observed in surviving rats [52]. In comparison, few studies have investigated the growth response upon excess iron provision in pre-weanling animals. We report that 90 mg iron/kg BW as FS is sufficient to disrupt growth in pre-weanling Lewis rat pups (Figure 1). Our finding that 10 mg iron/kg BW does not deleteriously impact weight gain in pre-weanling rats is consistent with prior literature [32]. Similarly, our group did not observe effects on weight gain with 30 or 150 ug per day as FS from PD 2–20—which equates to 0.6–3.8 mg iron/kg BW or 3.0–19 mg iron/kg BW, respectively [49,53]. Consistent with our results, Schröder et al. did not find weight gain effects when pre-weanling rats were provided 30 mg iron/kg BW as ferrous succinate [54]. Likewise, body weight gain in piglets provided 50 mg iron/kg BW daily was unaffected during the pre-weanling period [29]. In Holstein calves, weight gain was reduced for those formula containing 5000 mg iron/kg formula as FS (equating to approximately 500 mg iron/kg BW), but not with 2000, 1000, 500, or 100 mg iron/kg formula (~200, 100, 50, or 10 mg iron/kg BW) [55]. In our study, brain weight was reduced in the FS-90 group, but neither the FS-10 nor the FS-30 groups. We previously demonstrated that in pre-weanling Sprague Dawley rats, supplementation with FS-10 increased brain weight at PD 15 [32]. Together, these results suggest that growth and brain development outcomes upon iron supplementation depend on both dose and duration. Moreover, FS doses that disrupt normal growth in pre-weanling rat pups may be substantially lower than doses reported for adult rats. Additional studies are required to adequately define growth and mortality cutoffs for FS at this developmental stage.

In our FS dosing experiment, the liver was the tissue most affected by iron supplementation (Figure 2); staining for iron in liver sections revealed mixed iron loading, a type of iron loading that is observed with excess oral or parental iron supplementation, as well as late stage genetic hemochromatosis where iron granules are visible in hepatocytes, Kupffer cells, and portal macrophages [56]. Spleen and kidney iron levels increased moderately in the FS-30 and FS-90 groups, while iron levels in the brain were mostly similar between all groups. Of the four brain regions assessed, iron was increased in the striatum of FS-10 compared to CON; however, cortex, hippocampus, and cerebellum iron content were unaffected regardless of iron dose. With consideration to the reduced brain weight in the FS-90 group, it remains plausible that excess FS disrupts brain development without changing iron levels in major regions of the brain. Homeostatic mechanisms prevents iron loading in most extrahepatic tissues [43]. Hepatocytes store excess body iron and produce hepcidin, a hormone that protects against iron overload via suppressing intestinal iron absorption and reducing circulating iron release from the reticuloendothelial system [43]. Results presented here and previous radioisotope tracing experiments [49] provide evidence that hepatocyte iron regulation is intact in pre-weanling animals, where the liver accumulates excess dietary (absorbed) iron. Impaired growth observed in the FS-90 group may reflect altered liver function due to overt iron loading. However, histopathological evaluation of livers for inflammation, steatosis, fibrosis, and necrosis did not reveal pathological changes in liver tissue. Although we did not detect gross abnormalities in liver morphology, cellular and molecular changes, including alterations in genes involved in fibrotic possibilities are suggestive of alterations in tissue function due to excess iron. Future studies could seek to establish iron status cutoffs that predict the risk for injury in the liver and extrahepatic tissues, including the gastrointestinal tract where iron absorption occurs. 

Observed hemoglobin and hematocrit levels in CON animals were similar to other values reported at this age [57,58] and were not affected by excess iron at PD 10, in contrast to previous studies from our group [32,53,59]. The FS-30 group had higher MCH values than FS-10, but no significant effects were observed for any other RBC parameters (Table 1). [32]. Among the previous studies from our group that found increased hemoglobin with iron, only one measured hemoglobin at PD 10, whereas others assessed PD 15 and PD 21 [32,53,59]. Dubuque et al. observed no effect on hemoglobin, RBC, or MCV with iron at PD 12 [58]. RBC maturation and concentration increases with postnatal age [57], and thus effects of iron supplementation may be dynamic during the pre-weanling period. Pigs are at higher risk of anemia compared to other animal models and require iron injections for optimal growth during the pre-weanling period. Iron supplementation in piglets consistently increases hemoglobin and hematocrit in controlled experiments [29,60,61,62]. Conversely, studies involving iron supplementation of calves and foals have more mixed hematology results [55,63,64]. Variations in study design, including model species, iron dose, duration, and chemical form of supplement likely contribute to variability in iron assessment. Future studies should address whether exogenous iron provided in excess augments hematopoiesis in pre-weanling animals. In addition to established hematological parameters, assessing plasma iron and transferrin saturation levels in future studies would help clarify the availability of iron for RBC precursors.

Elevated WBC and neutrophil counts in the FS-90 group indicated a systemic inflammatory response upon excess iron exposure. Elevated serum CCL2, a pro-inflammatory cytokine, was observed in the FS-90 group and CCL2 has been shown to promote steatosis during liver injury [65]. Additionally, CXCL5 was reduced in FS-90 sera compared to CON. Lower levels of CXCL5 were observed in humans with chronic liver disease, and was associated with more severe necroinflammation and fibrosis [66]. Experiments in knockout mice have shown that Cxcl5 enhances neutrophil recruitment toward injured lung tissue by blocking pro-inflammatory chemokine scavenging from circulation [67]. Lower serum CX3CL1 was observed in both the FS-30 group and FS-90 group. CX3CL1 is upregulated in hepatocytes and hepatic stellate cells due to liver injury and becomes elevated in serum [68]. We did not measure hepatic CX3CL1 expression in the present study, and therefore cannot directly link our observations of lower serum concentrations with liver injury. To the best of our knowledge, no prior studies have reported serum chemokine and cytokine expression following excess iron at this developmental stage. 

Impairments in growth due to excess iron supplementation may be underscored by trace mineral interactions. Providing excess iron to infants may compromise their absorption and metabolism of other essential trace minerals [13,25,69,70]. As reported here (Figure 4), differential effects of excess iron on hepatic zinc and copper levels have been previously observed [49]. Liver zinc was elevated in all FS groups, with the highest levels observed in the FS-90 group. In contrast, liver copper levels were reduced only in the FS-90 group. In our prior study using pre-weanling Sprague Dawley rats, liver zinc was also elevated at PD 10 following daily supplementation with 30 or 150 µg iron per day FS, equating to 0.6–3.8 mg iron/kg BW or 3.0–19 mg iron/kg BW, respectively. Copper levels were reduced in the treatment group receiving the highest dose (3.0–19 mg iron/kg BW), while spleen and brain zinc and copper levels were unaffected [49]. Likewise, others have found lower serum and tissue copper levels as well as reduced weight gain in weanling rats fed very high levels of iron (9000 mg iron/kg diet as iron carbonyl) from PD 21–35; assuming 7–8 g food intake and 50–150 g BW [71], this equates to daily iron intake, approximately 500–1000 mg iron/kg BW daily intake [72]. Liver copper levels and weight gain were also reduced in weanling mice that consumed excess iron, attributed to impairments in copper utilization as opposed to absorption [73]. Moreover, others have observed elevated liver zinc and manganese in the absence of changes in liver copper [74]. Altered hepatic zinc and copper metabolism and storage may be the cause or result of local or systemic inflammatory signaling processes—potentiated by tissue iron loading [75,76].

Iron loading induces the expression of metallothionein (MT), a cellular zinc-binding protein that can also bind copper [77,78]. Specifically, iron upregulates Zip8 and Zip14, which preferentially transport zinc and iron at physiological pH [78]. Elevated hepatic zinc may reflect increased zinc import and binding by MT as a secondary consequence of tissue iron loading, a phenomenon that would be further exacerbated by iron toxicity-induced inflammatory signaling—compromising the availability of zinc for other growing tissues [77]. Likewise, lower levels of liver copper observed with increasing iron doses are likely a consequence of iron decreasing the uptake of copper by the liver, and interfere with its cellular roles, including mediation of reactive oxygen species as an enzyme cofactor [79]. In summary, impaired growth due to excess iron feeding may, to some degree, reflect disrupted zinc and/or copper metabolism.

Previous studies suggest that the effects of iron supplementation on postnatal development depend on sex [80,81]. Body weight and brain weight at the end of the study were negatively affected in FS-90 males but not in females. Spleen and kidney iron levels were also elevated in FS-90 males but not females. Liver zinc was elevated for FS males but not females; however, liver copper was reduced for both sexes in the FS-90 group. These results suggest male pups are more vulnerable to the deleterious effects of excess iron supplementation; however, the specific mechanism underlying these effects is unclear. To the best of our knowledge, no other study has tested the effect of sex on health risks of excess iron provision during a correlative developmental period. One possibility is that accelerated growth due to testosterone suppresses hepcidin and permits greater iron absorption, thereby heightening susceptibility to iron toxicity [82,83]. Direct measurements of testosterone are required to confirm the timing of mini-puberty in pre-weanling rats, and explore the possible interaction with hepcidin in early life. In adults, differences in iron requirements between sexes are largely due to the blood loss of menstruation, where males tend to have higher liver iron stores and hepcidin expression than females [81]. During infancy, a “mini-puberty” takes place: the hypothalamic-pituitary-gonadal (HPG) is activated leading to a surge of testosterone in male infants [84]. Testosterone has been shown to inhibit hepcidin transcription and stimulate iron absorption, which in the context of the current study may increase the risk of excess iron exposure [85]. In line with this hypothesis, growth acceleration during infancy has been linked to greater demand for iron and decreased hepcidin expression [86]. 

RNA-seq analysis of FS-supplemented livers identified genes that were powerfully upregulated in all FS group livers (Figure 5). Surprisingly, we did not observe changes in expression of MT nor ZIP genes; and, although there *Hamp* and *Tfrc*—genes involved iron regulation—were altered, the other transcripts were more strongly induced in FS livers. Additionally, *Tfrc* was downregulated only in FS-10 livers but not FS-30 or FS-90 livers, which was unexpected considering non-heme iron was elevated in all FS livers. *Itgb5*, which was up-regulated by over 1000-fold in all FS groups, encodes integrin beta 5, a protein involved liver fibrosis [87]. *Itgb5* was most strongly up-regulated in FS-10 and FS-30 livers (~1,000,000-fold CON expression). Although not observed histologically (i.e., fibrosis) by PD 10, up-regulation of *Itgb5* in livers of FS supplemented animals is suggestive of cellular dysregulation. *Rattus norvegicus* encode two *Itgb5* transcripts. Our results suggest differential regulation of *Itgb5* transcript variants in developing liver tissue in response to excess iron [39,88]. Forkhead box protein N3 (*Foxn3*), which is involved in regulation of hepatic glucose levels, was strongly induced in all FS groups but less-so in FS-90 livers. Overexpression of *Foxn3* is associated with elevated blood glucose and up-regulation of gluconeogenesis [89,90]. This suggests FS increased the activation of liver genes involved in increasing energy availability. Future studies might uncover important relationships between iron toxicity and growth by investigating energy metabolism in response to excess postnatal iron exposure.

The current study has several strengths, including supplementation design, dosing, and number of pups/litters assessed. Our findings support previous hypotheses that excess iron disrupts growth and trace mineral metabolism in pre-weanling animals and provide new data on brain iron and trace mineral loading effects, hematology, and chemokine/cytokine expression in response to iron provision. Excess daily iron supplementation at 90 mg iron/kg BW as FS disrupts weight gain in pre-weanling rats. Reduced growth was related to excess iron loading, but reduced brain weight was independent of brain iron levels. Iron loading in the liver following excess FS may induce tissue damage and inflammation as well as disrupt zinc and copper metabolism. Together, our results suggest liver damage, inflammation, and trace mineral interactions may be involved in the impaired growth outcomes of excess iron in pre-weanling rats. 

## Figures and Tables

**Table 1:** Complete blood counts.

	Group Mean ± Std. Deviation				
Result (Units) ^1^	CON	FS-10	FS-30	FS-90	*p*-Value ^2^
Hemoglobin (g/dL)	8.95 ± 0.44	8.50 ± 0.60	9.15 ± 0.25	9.03 ± 0.31	0.3635
Hematocrit (%)	31.0 ± 2.9	28.3 ± 1.5	30.8 ± 0.50	29.8 ± 3.3	0.4288
RBC (M/µL)	3.62 ± 0.17	3.52 ± 0.23	3.50 ± 0.11	3.50 ± 0.13	0.7836
MCV (fl)	85.5 ± 5.1	80.9 ± 1.4	87.7 ± 2.4	90.4 ± 0.85	0.1436
MCH (pg) ^2^	24.8 ± 0.17 ^a,b^	24.1± 0.25 ^a^	26.2 ± 0.22 ^b^	25.8 ± 0.15 ^a,b^	<0.0001, ****
MCHC (g/dL)	29.1 ± 1.8	29.8 ± 0.60	29.8 ± 0.71	28.6 ± 0.30	0.4185
RDW (%)	20.8 ± 2.2	18.0 ± 1.0	17.3 ± 0.50 ^†^	17.0 ± 1.0 ^†^	0.0080, **
WBC/µL	2938 ± 18	4170 ± 110	3403 ± 630	4210 ± 1300	0.1355
WBC/µL (corrected) ^3^	2874 ± 19 ^a^	3910 ± 77 ^a,b^	3300 ± 640 ^a,b^	4540 ± 620 ^b^	0.0095, **
Monocytes (%)	4.75 ± 2.8	6.00 ± 2.7	6.25 ± 2.6	7.25 ± 4.3	0.8003
Monocytes (count)	137 ± 78	244 ± 130	204 ± 81	310 ± 190	0.4937
Lymphocytes (%)	80.0 ± 3.7	78.3 ± 2.5	63.3 ± 30	68.3 ± 6.6	0.0976
Lymphocytes (count)	2300 ± 200	3060 ± 570	2150 ± 1200	2700 ± 610	0.3158
Neutrophils (%)	14.5 ± 2.1	15.0 ± 1.0	29.8 ± 30	23.0 ± 6.9	0.0689
Neutrophils (count)	416 ± 56	589 ± 130	924 ± 82	948 ± 480 ^†^	0.0486, *

^1^ RBC, red blood cells; MCV, mean cell volume; MCH, mean cell hemoglobin; MCHC, mean cell hemoglobin concentration; RDW, red blood cell distribution width; WBC, white blood cells. ^2^ Kruskal–Wallis test for effect of FS on mean, n = 3–4/group. *p*-value summary: *, *p* < 0.05; **, *p* < 0.01; ****, *p* < 0.0001. ^3^ Means with different superscripts are significantly different by post hoc Dunn’s test, *p* < 0.05. ^†^ Comparison to CON mean *p*-value: 0.08 > *p* > 0.05.

**Table 2:** Serum cytokine and chemokine protein concentrations.

Name of	Group Mean (pg/mL) ± Std. Deviation				
Protein	CON	FS-10	FS-30	FS-90	*p*-Value ^1^
G-CSF	23.5 ± 5.2	23.2 ± 5.9	23.8 ± 4.9	24.1 ± 8.7	0.9907
CCL11	5.44 ± 4.8	5.32 ± 3.9	5.91 ± 4.5	7.19 ± 5.3	0.8525
GM-CSF ^2^	9.87 ± 12 ^a,b^	0.00 ± 0.0 ^a^	3.98 ± 8.4 ^a,b^	17.4 ± 21 ^b^	0.0184, *
IL-1α	226 ± 200	164 ± 120	200 ± 74	289 ± 240	0.4027
Leptin^2^	39,800 ± 5500 ^a,b^	28,800 ± 9200 ^a^	55,500 ± 11,000 ^b^	46,300 ± 38,000 ^a,b^	0.0445, *
CCL3	63.5 ± 10	49.2 ± 7.7	58.2 ± 11	51.0 ± 18	0.0533
IL-4	0.770 ± 1.6	0.00 ± 0.0	0.385 ± 1.2	1.75 ± 3.2	0.2649
IL-1β	23.6 ± 7.2	18.8 ± 6.4	20.4 ± 8.7	144 ± 320	0.0594
IL-2	11.8 ± 14	18.2 ± 24	23.4 ± 12	24.5 ± 30	0.1524
IL-6	58.6 ± 190	0.00 ± 0.0	58.6 ± 190	319 ± 740	0.0856
EGF	37.2 ± 42	22.7 ± 20	14.8 ± 11	34.0 ± 42	0.7772
IL-13	0.669 ± 1.1	0.858 ± 1.9	1.20 ± 3.1	0.599 ± 1.9	0.7739
IL-10	78.0 ± 29	62.1 ± 22	55.0 ± 16	254 ± 540	0.2956
IL-12p70	22.7 ± 18	15.8 ± 14	32.7 ± 17	19.2 ± 28	0.1264
IFNγ	289 ± 110	209 ± 140	311 ± 95	180 ± 95 ^†^	0.0359, *
IL-5	31.9 ± 16	27.8 ± 17	43.1 ± 21	48.6 ± 26	0.1014
IL-17A	14.2 ± 2.7	11.6 ± 3.2	12.5 ± 2.6	14.1 ± 7.7	0.3261
IL-18	900 ± 320	987 ± 290	986 ± 400	1310 ± 400	0.0654
CCL2^2^	1640 ± 190 ^a,b^	1330 ± 190 ^a^	1640 ± 320 ^a,b^	1820 ± 620 ^b^	0.0274, *
IP-10	361 ± 40	343 ± 38	320 ± 60	341 ± 81	0.1078
CXCL1	145 ± 140	116 ± 140	153 ± 73	79.3 ± 100	0.3601
VEGF ^2^	211 ± 21 ^a^	192 ± 34 ^a,b^	167 ± 18 ^b^	193 ± 44 ^a,b^	0.0138, *
CX3CL1 ^2^	227 ± 18 ^a^	209 ± 22 ^a,b^	188 ± 22 ^b^	190 ± 25 ^b^	0.0018, **
CXCL5 ^2^	10,000 ± 2100 ^a^	8670 ± 1400 ^a,b^	8480 ± 1500 ^a,b^	7150 ± 1500 ^b^	0.0047, **
CXCL2 ^2^	24.2 ± 19 ^a^	4.46 ± 10 ^a,b^	0.00 ± 0.0 ^b^	18.1 ± 27 ^a,b^	0.0076, **
TNFα	4.94 ± 1.5	5.06 ± 1.2	4.58 ± 1.5	4.08 ± 1.1	0.3831
CCL5	57,800 ± 12,000	62,500 ± 19,000	52,800 ± 9100	49,200 ± 16,000	0.2522

^1^ Kruskal–Wallis or ANOVA test for effect of FS on mean, n = 10/group. *p*-value summary: *, *p* < 0.05; **, *p* < 0.01. ^2^ Means with different superscripts are significantly different by post hoc Dunn’s or Tukey’s test, *p* < 0.05. ^†^ Comparison to FS-30 mean *p*-value: 0.08 > *p* > 0.05.

## Data Availability

The data presented in this study are not publicly available. The data and full reproducible code are available on request from the corresponding author.

## Supplementary Materials

The following supporting information can be downloaded at: https://www.mdpi.com/article/10.3390/nu14193913/s1, Figure S1: Growth and development are disrupted only in male pups. Figure S2: Sex effects of excess FS on tissue iron loading; Figure S3: Sex effects on tissue zinc and copper concentrations; Table S1: Liver differential gene expression results from RNA-seq.
