# Supplementary material for: Trace Element Interactions, Inflammatory Signaling, and Male Sex Implicated in Reduced Growth Following Excess Oral Iron Supplementation in Pre-Weanling Rats
Source: Nutrients. 2022 Sep 21;14(19):3913. doi: 10.3390/nu14193913 (PMC9571796; doi:10.3390/nu14193913)
Supplement: Supplementary file 1 [file nutrients-14-03913-s001.zip › nutrients-1889775-supplementary.pdf]

Supplementary Information

Trace element interactions, inflammatory signaling, and male sex implicated in reduced growth following excess oral iron supplementation in pre-weanling rats

Shasta A. McMillen, Eric B. Nonnecke, and Bo Lönnerdal*

Supplementary Figure S1. Growth and development are disrupted only in male pups.
Supplementary Figure S2. Sex effects of excess FS on tissue iron loading.
Supplementary Figure S3. Sex effects on tissue zinc and copper concentrations.

Supplementary Table S1. Liver differential gene expression results from RNA-seq.

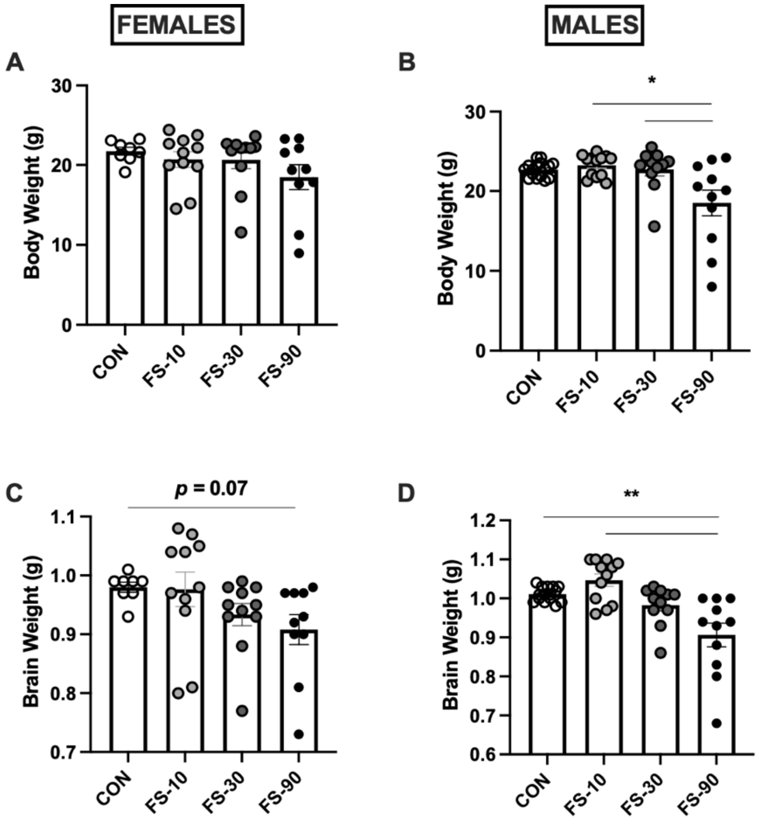

Supplementary Figure S1. Growth and development are disrupted only in male pups that received excess FS. (A-B) Body weight is reduced in FS-90 males but not females at study end point, PD 10. (C-D) Brain weight at necropsy on PD 10 is reduced in males but females that received excess FS. Means ± SEM are shown. *p*-value summary: *, $p < 0.05$; **, $p < 0.01$.

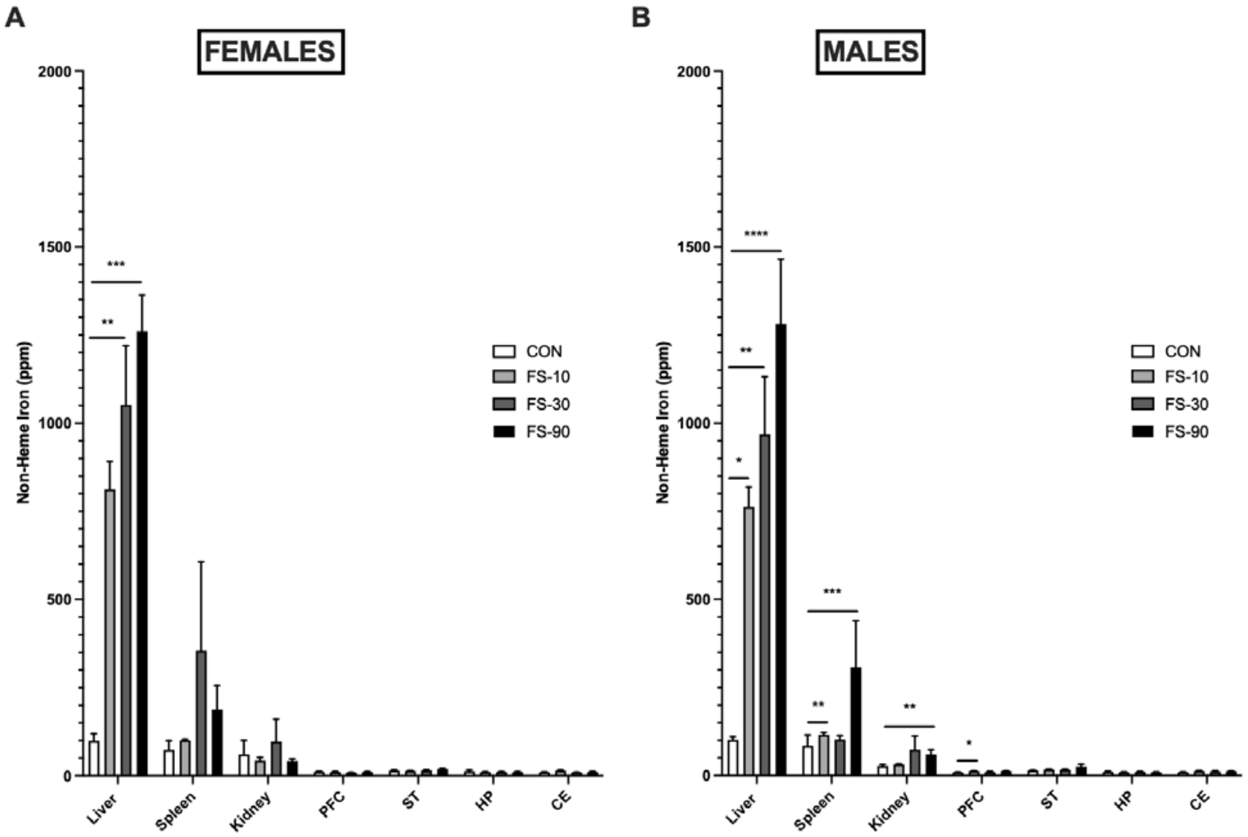

Supplementary Figure S2. Sex effects of excess FS on tissue iron loading (n = 7-13/group*sex for each tissue). Means ± SEM are shown. *p*-value summary: *, $p < 0.05$; **, $p < 0.01$; ***, $p < 0.001$; ****, $p < 0.0001$.

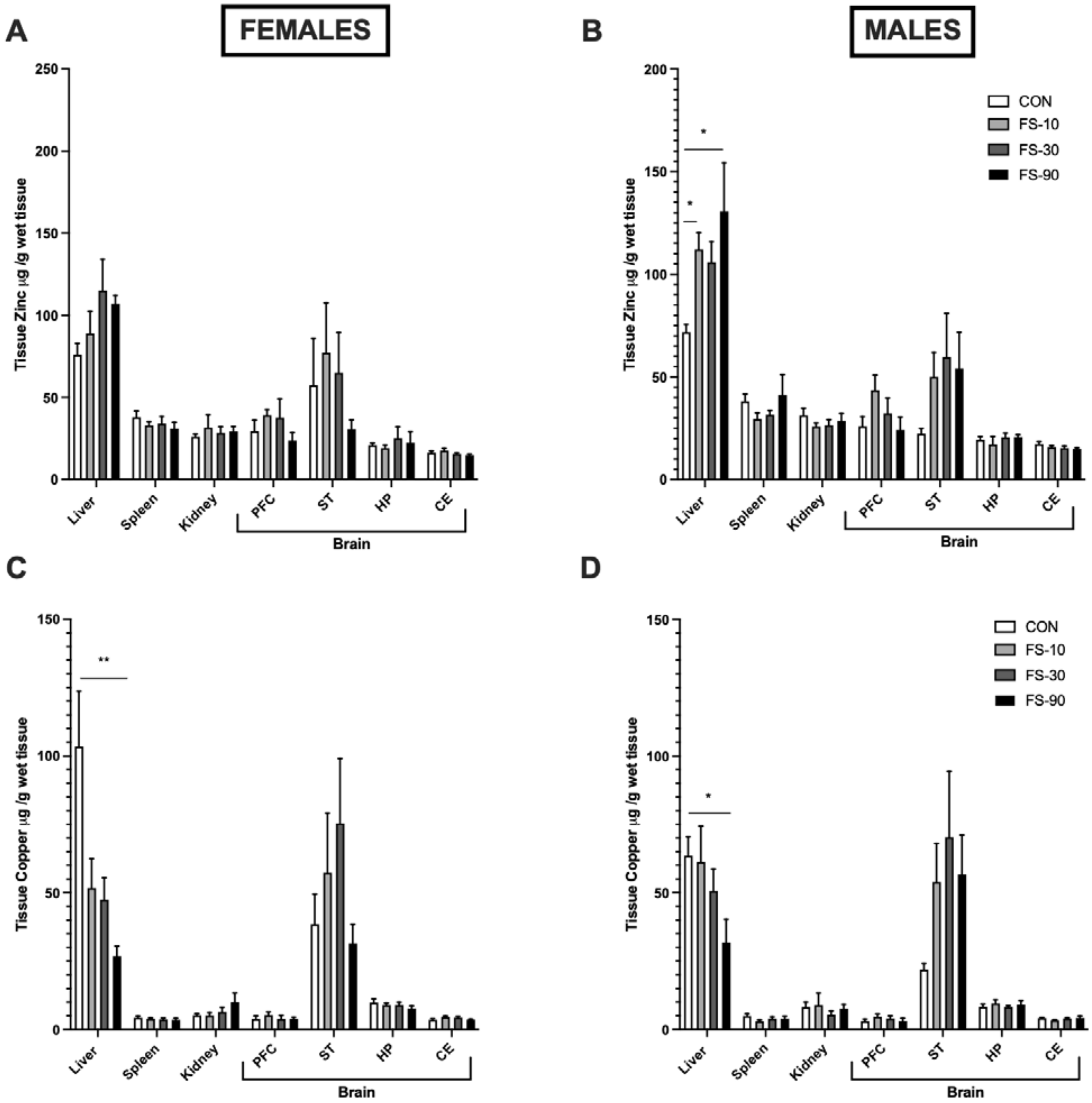

Supplementary Figure S3. Sex effects of excess FS on tissue zinc and copper (n = 4-10/group*sex for each tissue). Means ± SEM are shown. *p*-value summary: *, $p < 0.05$; **, $p < 0.01$.

Supplementary Table S1. Liver differential gene expression results from RNA-seq.

Symbol	Gene	Comparison[1]	Base Mean	log_2[FoldChange]	lfcSE	padj
Psma1	ENSRNOT00000015946	FS-30	99.3694709	-23.90797968	4.24747384	4.62E-05
Ugta3	ENSRNOT00000071200	FS-30	97.1128259	-22.06492987	3.29068547	1.02E-07
Dmxl1	ENSRNOT00000031812	FS-30	21.610229	-21.70413453	4.11240444	0.00026607
Ybx3	ENSRNOT00000068185	FS-90	44.3919631	-21.69207577	4.24923763	0.00021844
Itgb5	ENSRNOT00000076955	FS-90	79.4542088	-9.786700723	1.58018634	5.18E-07
Cwc15	ENSRNOT00000011855	FS-10	148.742132	-9.703570843	1.72805349	3.93E-05
Tfrc	ENSRNOT00000002407	FS-10	191.216796	-2.944836987	0.65962815	0.00602732
Slc25a39	ENSRNOT00000028496	FS-90	515.153019	0.428443225	0.10035728	0.00545315
Hsp90ab1	ENSRNOT00000086986	FS-90	1396.06066	0.728958285	0.16731845	0.0041015
Pgrmc1	ENSRNOT00000017101	FS-90	753.687662	1.016366449	0.13983535	4.81E-10
Gsta3	ENSRNOT00000088416	FS-90	533.622679	1.104091596	0.23828402	0.00146046
Ifi27l2b	ENSRNOT00000032015	FS-90	125.371	1.221764785	0.22711818	5.64E-05
Creg1	ENSRNOT00000004394	FS-90	222.510467	1.370311179	0.28464931	0.00070878
Reep6	ENSRNOT00000044030	FS-90	58.1866938	1.611781016	0.38314882	0.00684312
Id1	ENSRNOT00000029660	FS-90	68.7151685	1.861341821	0.28831734	1.14E-07
Ephx1	ENSRNOT00000085279	FS-90	424.766402	2.042126616	0.47708024	0.00545315
Gstt3	ENSRNOT00000036518	FS-90	422.640076	2.900036775	0.34150673	3.58E-14
Orm1	ENSRNOT00000010454	FS-90	76.2743778	4.408184664	1.00074751	0.00372615
Hamp	ENSRNOT00000028545	FS-10	1225.29613	4.593018754	0.73581391	1.30E-06
Hamp	ENSRNOT00000028545	FS-30	1225.29613	4.663439979	0.73578556	7.89E-07
Hectd1	ENSRNOT00000008459	FS-10	13.4937851	7.056688318	1.56251775	0.00540026
Hectd1	ENSRNOT00000008459	FS-90	13.4937851	7.060535756	1.56311131	0.00236623
Hamp	ENSRNOT00000028545	FS-90	1225.29613	7.251512621	0.73514417	1.57E-19
Foxn3	ENSRNOT00000006604	FS-90	23.4720857	18.64156922	4.25332759	0.00386678
Sash1	ENSRNOT00000017688	FS-30	19.2409961	19.50994063	4.25036112	0.00643468
Itgb5	ENSRNOT00000049974	FS-30	64.6317126	19.62129134	2.09514658	7.73E-17
Ivns1abp	ENSRNOT00000087997	FS-10	14.3500605	19.64007132	3.90351316	0.00073104
Ivns1abp	ENSRNOT00000087997	FS-90	14.3500605	19.7370125	3.90350675	0.00025093
Foxn3	ENSRNOT00000006604	FS-30	23.4720857	20.12580448	4.24917172	0.00368768
Dctn2	ENSRNOT00000008120	FS-10	20.8964227	20.17541834	4.24947664	0.00205872
Foxn3	ENSRNOT00000006604	FS-10	23.4720857	20.23088374	4.24899609	0.00205872
Sash1	ENSRNOT00000017688	FS-90	19.2409961	20.38223564	4.24895016	0.00070878
Dctn2	ENSRNOT00000008120	FS-90	20.8964227	20.76787809	4.24876393	0.000538
Itgb5	ENSRNOT00000049974	FS-10	64.6317126	21.57672123	2.09081074	3.44E-21
Itgb5	ENSRNOT00000049974	FS-90	64.6317126	21.66084526	2.09082094	1.99E-21

[1]Group compared to CON in differential gene expression analysis
